# An Intuitive End-to-End Human-UAV Interaction System for Field Exploration

**DOI:** 10.3389/fnbot.2019.00117

**Published:** 2020-02-14

**Authors:** Ran Jiao, Zhaowei Wang, Ruihang Chu, Mingjie Dong, Yongfeng Rong, Wusheng Chou

**Affiliations:** ^1^School of Mechanical Engineering and Automation, Beihang University, Beijing, China; ^2^College of Mechanical Engineering and Applied Electronics Technology, Beijing University of Technology, Beijing, China; ^3^The State Key Laboratory of Virtual Reality, Technology and Systems, Beihang University, Beijing, China

**Keywords:** UAV, intuitive interaction, pose estimation, super-twisting, extended state observer, back-stepping

## Abstract

This paper presents an intuitive end-to-end interaction system between a human and a hexacopter Unmanned Aerial Vehicle (UAV) for field exploration in which the UAV can be commanded by natural human poses. Moreover, LEDs installed on the UAV are used to communicate the state and intents of the UAV to the human as feedback throughout the interaction. A real time multi-human pose estimation system is built that can perform with low latency while maintaining competitive performance. The UAV is equipped with a robotic arm, kinematic and dynamic attitude models for which are provided by introducing the center of gravity (COG) of the vehicle. In addition, a super-twisting extended state observer (STESO)-based back-stepping controller (BSC) is constructed to estimate and attenuate complex disturbances in the attitude control system of the UAV, such as wind gusts, model uncertainties, etc. A stability analysis for the entire control system is also presented based on the Lyapunov stability theory. The pose estimation system is integrated with the proposed intelligent control architecture to command the UAV to execute an exploration task stably. Additionally, all the components of this interaction system are described. Several simulations and experiments have been conducted to demonstrate the effectiveness of the whole system and its individual components.

## 1. Introduction

UAVs, which have been increasingly used as human assistants in various contexts in recent years, are developing very rapidly. They can be applied in areas to which humans cannot reach, such as for aerial photography, field exploration, etc. Also, human-robot interaction (Fang et al., 2019) has also been focused on recently, including human-UAV interaction technology. However, a traditional approach to the interaction between UAVs equipped with remote devices and a human is not convenient when that human is busy with other tasks during field exploration. This paper aims to build an intuitive end-to-end human-UAV interaction system for field exploration where mutual attention between the human and UAV is established in the process.

The interface used to control UAVs is an important part of the whole interaction system. It can be classified into two kinds, traditional human-computer interfaces and direct interfaces. As to the former, Rodriguez et al. (2013) designed ground control station software that is fully based on open-source libraries and developed it for a platform composed of multiple UAVs for surveillance missions. Moreover, utility software designed by McLurkin et al. (2006) for interacting with hundreds of autonomous robots without having to handle them individually enables centralized development and debugging. In addition, several principles of swarm control are studied in Kolling et al. (2012) and are used in a simulated robot environment to enable a human operator to impose on and control large swarms of robots. Of the direct interfaces, many of them have been applied in human-UAV interaction systems in recent years. Pourmehr et al. (2013b) presents a multi-model system to create, modify, and command groups of robots, in which groups of robots can be created by speaking their numbers. Additionally, a whole system in which multiple humans and robots could interact with each other using a combination of sensing and signaling modalities was built by Pourmehr et al. (2013a). In our work, we use the direct interaction mode for the design of a natural and intuitive human-UAV interaction system as an assistant for field exploration. Similar to the interaction system mentioned by Monajjemi et al. (2013), human poses are used to give commands to the UAV in our interaction system. Therefore, the human detection system should be built first.

We intend to use several different natural human poses to communicate with the UAV. Previous research has looked into detecting serial human poses. A method based on Lagrangian particle trajectories, which are a suite of dense trajectories obtained by advecting optical flow over time, is proposed to capture the ensemble motions of a scene by Wu et al. (2011). Moreover, Bin et al. (2018) proposes a novel data glove for pose capturing and recognition based on inertial and magnetic measurement units (IMMUs). Additionally, Ran et al. (2007) proposes two related strategies. The first estimates a periodic motion frequency with two cascading hypothesis testing steps to filter out non-cyclic pixels, and the second involves converting the cyclic pattern into a binary sequence by fitting the Maximal Principal Gait Angle. Pishchulin et al. (2016) proposes a method to jointly solve the tasks of detection and pose estimation in which the number of persons in a scene can be inferred, occluded body parts can be identified, and body parts between people in close proximity of each other can also be disambiguated. However, it cannot be performed with low latency and cannot be applied in an embedded device and used for a UAV.

Once the human pose is detected, under the control of the human pose and referring to the interaction regulation scheme developed in this paper, the UAV would respond and approach the human for further particular commands. However, the UAV's positional motion is coupled with rotary movement, and both of them can be influenced easily. When performing tasks, it is normal for a UAV to encounter wind gust disturbance, which would affect the stability of the whole system. Moreover, to carry out exploration tasks that may be encountered in the future, the UAV is equipped with a 2-DOF robotic arm, which would bring more model uncertainties to the overall system. The disturbance estimation and attenuation are thus the next problem to overcome. Several similar works have been carried out, such as on disturbance and uncertainty estimation and attenuation (DUEA) strategy, which has been widely used and explored in recent years (Yang et al., 2016). Also, numerous observers have been designed to solve this problem, for example, a disturbance observer (DO) (Zhang et al., 2018; Zhao and Yue, 2018) and extended state observer (ESO) (Shao et al., 2018). Moreover, Mofid and Mobayen (2018) proposes a technique of adaptive sliding mode control (ASMC) for finite-time stabilization of a UAV system with parametric uncertainties. Additionally, a higher-order EDO was applied for attitude stabilization of flexible spacecraft while investigating the effects of different orders on the performance of the EDO (Yan and Wu, 2017). It has been proved that the estimation accuracy can be improved with an increase in the observer order via choosing suitable observer gains. Nevertheless, a higher order of the observer will lead to both high implementation cost and the problem of high gain for observers.

In this paper, an intuitive, natural, end-to-end human-UAV interaction system is built for field exploration assistance. The entire attitude dynamic model of the hexacopter UAV equipped with a robotic arm is presented considering the robotic arm as an element affecting the COG of the vehicle. Moreover, through replacing the backbone network VGG-19 in Cao et al. (2017) by the first twelve layers of MobileNetV2, a real time multi-human pose estimation system, which can be performed with lower latency, maintaining the competitive performance, is built for humans to communicate with the UAV under a proposed interaction regulation. Both target flight direction and distance commands can be transmitted to the UAV easily and naturally. In addition, as a UAV equipped with a robotic arm has more model uncertainty than traditional UAVs and wind gust cannot usually be avoided when carrying out exploration tasks, a composite controller is designed by combining STESO (Shi et al., 2018b) and a back-stepping control method. As most of the disturbances, including wind gust and model uncertainties, are compensated by the feedforward compensator based on STESO, only a small switching gain is required in the controller. Thus, high-accuracy UAV attitude tracking can be realized, and chattering can be alleviated in the presence of several disturbances. Moreover, depth estimation with a binocular camera was developed according to the work of Zhang (2000). The effectiveness of the proposed interaction system and its individual components is demonstrated in several simulations and experiments.

The outline of this work is as follows. Some preliminaries, including quaternion operations and the kinematic and dynamic attitude models of the whole hexacopter UAV are presented in section 2. In section 3, several methods such as human pose estimation, depth estimation, STESO construction, attitude controller, and interaction regulation scheme are formulated. Several simulations and experiments are then given in sections 4 and 5, respectively. Finally, the conclusion is summarized in section 6.

## 2. Preliminaries

### 2.1. Notation

The maximal and minimum eigenvalues of matrix H are given by λ_max_(H) and λ_min_(H), respectively, and ||·|| represents the 2-norm of a vector or a matrix. Additionally, the operator ***S*(·)** denotes a vector $\kappa ={\left[\begin{array}{ccc} \kappa_{1} & \kappa_{2} & \kappa_{3} \end{array}\right]}^{T}$ to a skew symmetric matrix as:

(1) $$\begin{array}{r} \text{S}\left(\kappa \right)=\left[\begin{array}{ccc} 0 & -\kappa_{3} & \kappa_{2}\\ \kappa_{3} & 0 & -\kappa_{1}\\ -\kappa_{2} & \kappa_{1} & 0 \end{array}\right] \end{array}$$

The sign function can be described as:

(2) $$\begin{array}{r} sign\left(\kappa \right)=\{\begin{array}{c} \frac{\kappa }{\left|\kappa \right|},|\kappa |\neq 0\\ 0,|\kappa |=0 \end{array} \end{array}$$

### 2.2. Quaternion Operations

As traditional methods used for representing rotation of the UAV, for instance, the Euler angles, may lead to the singularity problem of trigonometric functions, the unit quaternion $\text{q}={\left[q_{0}{\text{q}}_{v}\right]}^{T}\in {\text{R}}^{4}$, ||***q***|| = 1 is utilized in this work Shastry et al. (2018). Several corresponding operations are defined as follows.

The quaternion multiplication:

(3) $$\begin{array}{r} \text{q}⊗\sigma =\left[\begin{array}{c} q_{0}\sigma_{0}-{\text{q}}_{\text{v}}^{\text{T}}\sigma_{\text{v}}\\ q_{0}\sigma_{\text{v}}+\sigma_{0}{\text{q}}_{\text{v}}-\text{S}\left(\sigma_{\text{v}}\right){\text{q}}_{\text{v}} \end{array}\right] \end{array}$$

The relationship between rotation matrix ${\text{C}}_{\text{A}}^{\text{B}}$ and unit quaternion ***q*** is described as:

(4) $$\begin{array}{r} {\text{C}}_{\text{A}}^{\text{B}}=\left(q_{0}^{2}-{\text{q}}_{\text{v}}^{\text{T}}{\text{q}}_{\text{v}}\right){\text{I}}_{3}+2{\text{q}}_{\text{v}}{\text{q}}_{\text{v}}^{\text{T}}+2q_{0}\text{S}\left({\text{q}}_{\text{v}}\right) \end{array}$$

The time derivative of Equation (4) is:

(5) $$\begin{array}{r} \dot{{\text{C}}_{\text{A}}^{\text{B}}}=-\text{S}\left(\omega \right){\text{C}}_{\text{A}}^{\text{B}} \end{array}$$

where the details of coordinate systems ***A*** and ***B*** will be given in the next section. Then, the derivative of a quaternion and the quaternion error ***q*_*e*_** are given as follows, respectively:

(6) $$\begin{array}{r} \dot{\text{q}}=\left[\begin{array}{c} {\dot{q}}_{0}\\ {\dot{\text{q}}}_{v} \end{array}\right]=\frac{1}{2}\text{q}⊗\left[\begin{array}{c} 0\\ \omega \end{array}\right]=\frac{1}{2}\left[\begin{array}{c} -{\text{q}}_{\text{v}}^{T}\\ \text{S}\left({\text{q}}_{\text{v}}\right)+q_{0}{\text{I}}_{3} \end{array}\right]\omega \end{array}$$

(7) $$\begin{array}{rcl} {\text{q}}_{\text{e}} & = & {\text{q}}_{\text{d}}^{*}⊗\text{q} \end{array}$$

where ***q*_*d*_** denotes the desired quaternion whose conjugate is represented by ${\text{q}}_{\text{d}}^{*}={\left[q_{d0}-{\text{q}}_{\text{d}\text{v}}\right]}^{T}$, **ω** is the angular velocity of the system.

### 2.3. Kinematic and Dynamic Models of Hexacopter UAV

**Figure 1:**
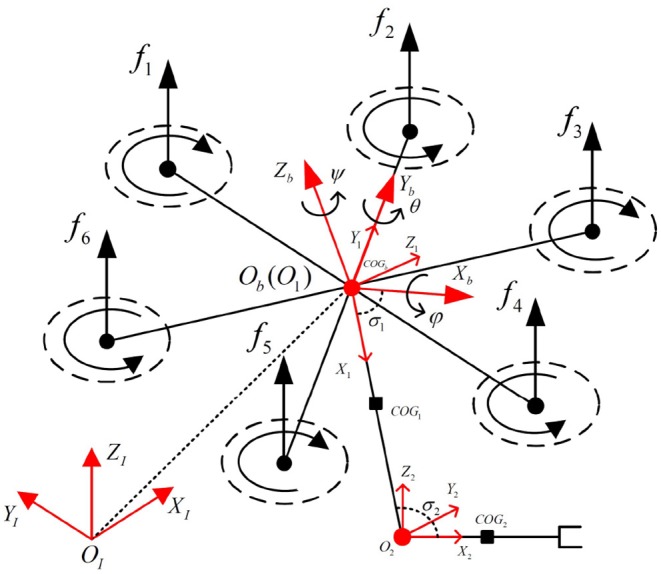
Illustration of the hexacopter and robotic arm system with related coordinate reference frames.

As depicted in Figure 1, The whole UAV system used for interaction with humans is a hexacopter equipped with a 2-DOF robotic arm. The robotic arm is fixed at the geometric center of the hexacopter. The kinematic and dynamic models of the system are detailed below.

#### 2.3.1. Kinematic Model

The kinematic model of the UAV system can be achieved with several related reference coordinates in Figure 1, which are defined as follows:

*O*_*I*_: world-fixed inertial reference frame

*O*_*b*_: hexacopter body-fixed reference frame located at the geometric center of the vehicle

*O*_*d*_: desired reference frame located at the geometric center of the vehicle

*O*_*i*_: frame fixed to link *i* in the robotic arm. *i* = {1, 2}.

Additionally, several coefficients are used to describe the overall system. **Γ** = [*x, y, z*]^*T*^ represents the absolute position of *O*_*b*_ with reference to *O*_*I*_. The UAV attitudes are described by Euler angles **Ψ** = [φ, θ, ψ]^*T*^ whose components represent roll, pitch, and yaw angles, respectively. In addition, the absolute linear velocity of the hexacopter with respect to *O*_*b*_ is denoted by $\text{V}={\left[v_{x},v_{y},v_{z}\right]}^{T}$, and $\omega ={\left[\omega_{x},\omega_{y},\omega_{z}\right]}^{T}$ represents the vector of the absolute rotational velocity of the hexacopter with respect to *O*_*b*_. The relation can be described as:

(8) $$\begin{array}{r} \omega ={\text{R}}_{r}\dot{\text{Ψ}} \end{array}$$

where

(9) $$\begin{array}{r} {\text{R}}_{r}=\left[\begin{array}{ccc} 1 & 0 & -\text{s}\theta \\ 0 & \text{c}\varphi & \text{s}\varphi \text{c}\theta \\ 0 & -\text{s}\varphi & \text{c}\varphi \text{c}\theta \end{array}\right] \end{array}$$

where *c*(·) and *s*(·), mentioned above, are the abbreviations of *cos*(·) and *sin*(·).

#### 2.3.2. Dynamic Model

A traditional UAV with a constant COG at its geometrical center can be described with simple dynamic model equations (Bouabdallah and Siegwart, 2005). However, the motion of a robotic arm will affect the position of the vehicle. To consider the robotic arm as an element leading to the displacement of the COG from the geometric center of the vehicle, a dynamic attitude model of the whole system is provided in this subsection. Referring to our previous work Jiao et al. (2018), it can be given as:

(10) $$\begin{array}{r} \{\begin{array}{c} J_{x}{\dot{\omega }}_{x}=u_{1}-\left(J_{z}-J_{y}\right)\omega_{y}\omega_{z}-mc_{1}+d_{x}\\ J_{y}{\dot{\omega }}_{y}=u_{2}-\left(J_{x}-J_{z}\right)\omega_{x}\omega_{z}-mc_{2}+d_{y}\\ J_{z}{\dot{\omega }}_{z}=u_{3}-\left(J_{y}-J_{x}\right)\omega_{x}\omega_{y}-mc_{3}+d_{z} \end{array} \end{array}$$

where

(11) $$\begin{array}{r} \{\begin{array}{c} c_{1}=y_{G}\left({\dot{v}}_{z}-v_{x}\omega_{y}+v_{y}\omega_{x}\right)-z_{G}\left({\dot{v}}_{y}-v_{z}\omega_{x}+v_{x}\omega_{z}\right)\\ c_{2}=-x_{G}\left({\dot{v}}_{z}-v_{x}\omega_{y}+v_{y}\omega_{x}\right)+z_{G}\left({\dot{v}}_{x}-v_{y}\omega_{z}+v_{z}\omega_{y}\right)\\ c_{3}=-y_{G}\left({\dot{v}}_{x}-v_{y}\omega_{z}+v_{z}\omega_{y}\right)+x_{G}\left({\dot{v}}_{y}-v_{z}\omega_{x}+v_{x}\omega_{z}\right) \end{array} \end{array}$$

We describe Equation (11) in a collective form:

(12) $$\begin{array}{l} \text{J}\dot{\omega }=\text{u}-\text{S}\left(\omega \right)\text{J}\omega -m\text{c}+\text{d} \end{array}$$

where vector ***J*** = *diag*(*J*_*x*_, *J*_*y*_, *J*_*z*_) indicates that the inertia matrix is diagonal, and $\text{d}={\left[d_{x},d_{y},d_{z}\right]}^{T}$ denotes the lumped disturbances caused by wind gusts, model uncertainties, etc. The COG of the whole UAV system is described by ${\text{C}}_{\text{G}}={\left[x_{G},y_{G},z_{G}\right]}^{T}$. *m* is the total mass of the UAV. Additionally, we define vector $\text{c}={\left[c_{1},c_{2},c_{3}\right]}^{T}$ and vector $\text{u}={\left[u_{1},u_{2},u_{3}\right]}^{T}$, representing the control torque inputs, in which the torques around *x*−, *y*−, and *z*− generated by the six propellers are represented by *u*_1_, *u*_2_, and *u*_3_, respectively. This has the following expression:

(13) $$\begin{array}{r} \text{u}=\Xi {\text{f}}_{\text{v}} \end{array}$$

where ${\text{f}}_{\text{v}}={\left[\omega_{1}^{2},\omega_{2}^{2},\omega_{3}^{2},\omega_{4}^{2},\omega_{5}^{2},\omega_{6}^{2}\right]}^{T}$ represents a positive correlation vector with forces generated from the hexacopter motors, in which ω_*i*_ denotes the rotor speed of the hexacopter (*i* = 1, 2, 3, 4, 5, 6). In addition, referring to the hexacopter model in Figure 1, **Ξ** can be expressed as follows:

(14) $$\begin{array}{r} \Xi =\left[\begin{array}{cccccc} \frac{l}{2}\Lambda_{T} & l\Lambda_{T} & \frac{l}{2}\Lambda_{T} & -\frac{l}{2}\Lambda_{T} & -l\Lambda_{T} & -\frac{l}{2}\Lambda_{T}\\ -\frac{\sqrt{3}}{2}l\Lambda_{T} & 0 & \frac{\sqrt{3}}{2}l\Lambda_{T} & \frac{\sqrt{3}}{2}l\Lambda_{T} & 0 & -\frac{\sqrt{3}}{2}l\Lambda_{T}\\ \Lambda_{C} & -\Lambda_{C} & \Lambda_{C} & -\Lambda_{C} & \Lambda_{C} & -\Lambda_{C} \end{array}\right] \end{array}$$

where Λ_*T*_ and Λ_*C*_ denote the thrust and drag coefficients, respectively. Moreover, *l* represents the distance from each motor to the center of mass of the hexacopter.

## 3. Methods

### 3.1. Human Pose Estimation

Human pose estimation is a prerequisite component of the human-UAV interaction system. It efficiently detects the 2D poses of people in an image. The pose information serves as the coded target within the human-UAV communication, in which each pose form is designed as a special command, guiding the UAV to perform desired tasks.

The challenges of human pose estimation are two-fold. First, under uncertainties, each image may contain multiple people in various positions and at different scales. Vision-based pose estimation may easily suffer from distraction by irrelevant people, which requires us to design an identification algorithm. It must ignore the non-target candidate people and thus choose the right commander. Second, the above-mentioned commander identification is under the premise that all candidate people can be detected. If we equip each person with a pose detector, the runtime is proportional to the number of people. This would bring significant latency and severely deteriorate the stability of interaction.

To build a time-consuming multi-human pose estimation system, we follow Cao et al. (2017) to employ a bottom-up pose predictor, which means that part locations are first detected and then associated to limbs. Unlike top-down approaches that infer the limb based on each person detection, the bottom-up approach decouples time complexity from the number of people. Specifically, we adopt a two-branch neural network to learn part locations and their associations, respectively. Both of them contribute to the subsequent multi-person parsing process.

The network architecture remains the same as that in Cao et al. (2017), in which an image is taken as input and the connected limbs, i.e., poses, of multiple people are outputs. The raw image first passes through a stack of convolutional layers, generating a set of feature maps. In this stage, we replace VGG-19 (Simonyan and Zisserman, 2014) by the first twelve layers of MobileNetV2 (Sandler et al., 2018) to make it more lightweight, as VGG-19 results in large computational costs and repeatedly employs small-size (3 × 3) convolutional filters to enhance network capacity. In contrast to VGG-19, MoblieNetV2 adopts a novel depthwise separable convolution to reduce actual latency while maintaining competitive performance. The feature maps can be regarded as deep semantic representations of the image, which are then fed into two convolutional branches. The confidence maps and part affinity fields are produced from two branches in parallel. The confidence map predicts the possibility that a particular part occurs at each pixel location, and the part affinity fields measure the confidence of part-to-part association. Finally, the network implements multi-person parsing, which assembles the parts to form the full-body poses of all of the people.

**Figure 2:**
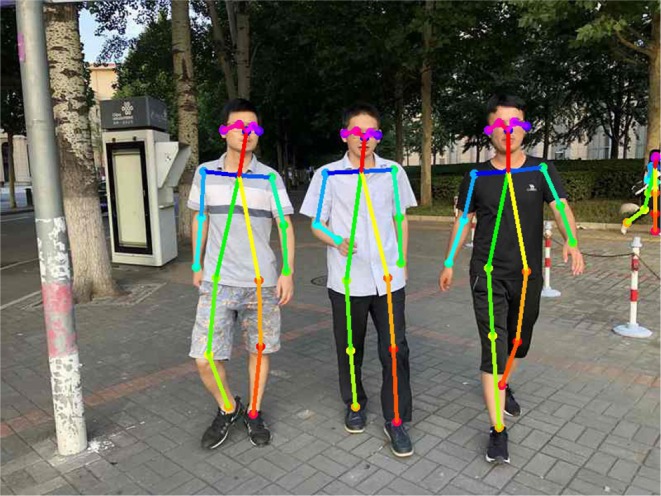
Result of the human pose-estimation system. Written informed consent for publication was obtained from the individuals in this image.

Through this pipeline, multi-human pose estimation can be performed with low latency. The time efficiency is derived not only from the bottom-up inference approach but also from the backbone network used. The bottom-up inference makes run time irrespective of the number of people, allowing the potential for real-time multi-human pose estimation. The selected MobilenetV2 further reduces the number of operations during inference by avoiding large intermediate tensors. To investigate the performance, we train our network on an MPII Multi-person dataset (Andriluka et al., 2014) and test it on our own datasets. During training, the image is resized to (432 × 368). We apply the Adam optimizer (Kingma and Ba, 2014) with default settings $\left(\varepsilon =10^{-3},\beta_{1}=0.9,\beta_{2}=0.999\right)$. The learning rate is set to 0.001, and the batch size is 64. The result for the human image is shown in Figure 2. It can be clearly seen that all human poses are correctly detected. Notably, our system achieves a frame-rate of about 6 fps running on an NVIDIA TX2 and, when we adopt the VGG-19 as the backbone, the frame-rate drops to about 2 fps. This proves the suitability of MobileNetV2 for mobile applications, especially our human-UAV interaction system.

### 3.2. Depth Estimation

The depth estimation for the camera installed on the UAV is conducted using a binocular stereo vision ranging method, which is composed of four main parts, namely camera calibration, stereo calibration, stereo rectification, and image matching. The internal and external parameters of the camera are obtained in the camera calibration step, referring to the method of Zhang (2000). Stereo calibration is performed to get the pose and position of one camera with respect to the other. In addition, stereo rectification is used to align image rows between two cameras. The disparity value, which is essential for determining the distance between object and camera, can then be obtained through only searching one row in the image matching step for a match with a point in the other image after the target point is determined. Obviously, this will enhance computational efficiency. Both the stereo rectification and image matching steps are conducted with the use of OpenCV functions. Then, referring to Xuezhi (2014), the depth can be obtained after several works mentioned above.

### 3.3. Super Twisting Extended State Observer (STESO)

The UAV equipped with a robotic arm has more model uncertainty than a traditional UAV. Moreover, other external disturbances such as wind gusts cannot usually be avoided when carrying out exploration tasks. In this section, all of the disturbances exerted on a UAV are seen as a lumped disturbance, and a STESO is built to estimate it in finite time.

The accelerated velocities $\dot{\text{v}}$ and angular velocities **ω** can be measured by a MEMS accelerometer and gyroscope, respectively, and the lateral velocities can be obtained directly from GPS. Regarding the dynamics (Equation 12) of the whole UAV system, by importing the feedback linearization method, the original control input can be reformulated as:

(15) $$\begin{array}{r} \text{u}={\text{u}}^{*}+\text{S}\left(\omega \right)\text{J}\omega +m\text{c} \end{array}$$

The linearized dynamic model can then be given as:

(16) $$\begin{array}{r} \text{J}\dot{\omega }={\text{u}}^{*}+\text{d} \end{array}$$

When building the STESO, it is assumed that each channel is independent, so only one portion is introduced in this subsection and the other two are completely identical. Regarding Equation (16), the one-dimensional dynamics of the UAV used for building the STESO is given as:

(17) $$\begin{array}{r} J_{i}{\dot{\omega }}_{i}=u_{i}^{*}+d_{i} \end{array}$$

By importing a new extended state vector $\zeta_{i}={\left[\zeta_{i,1},\zeta_{i,2}\right]}^{T}$, in which ζ_*i*, 1_ = *J*_*i*_ω_*i*_ and ζ_*i*, 2_ = *d*_*i*_,(*i* = *x, y, z*), the original dynamic model can be constructed as follows:

(18) $$\begin{array}{r} \{\begin{array}{c} {\dot{\zeta }}_{i,1}=u_{i}^{*}+\zeta_{i,2}\\ {\dot{\zeta }}_{i,2}=\chi_{i} \end{array} \end{array}$$

where χ represents the derivative of *d*_*i*_ and it is assumed that |χ| < ν^+^, meaning that the lumped disturbance, is bounded.

As the system Equation (18) is observable, the STESO can be designed for this system by introducing a super-twisting algorithm (Yan and Wu, 2019):

(19) $$\begin{array}{r} \{\begin{array}{c} {\dot{z}}_{1}=z_{2}+u_{i}^{*}+\xi_{1}{\left|e_{1}\right|}^{\frac{1}{2}}sign\left(e_{1}\right)\\ {\dot{z}}_{2}=\xi_{2}sign\left(e_{1}\right) \end{array} \end{array}$$

where *z*_1_ and *z*_2_ represent estimates of ζ_*i*, 1_ and ζ_*i*, 2_, respectively. $e_{1}=\zeta_{i,1}-{\hat{\zeta }}_{i,1}$ and $e_{2}=\zeta_{i,2}-{\hat{\zeta }}_{i,2}$ are estimate errors. The whole system estimate errors *e*_1_ and *e*_2_ can be ensured to converge to zero within finite time with appropriate observer gains ξ_1_ and ξ_2_.

***Proof*.** According to Equations (18) and (19), the error dynamics of the STESO can be obtained as:

(20) $$\begin{array}{r} \{\begin{array}{c} {\dot{e}}_{1}=e_{2}-\xi_{1}{\left|e_{1}\right|}^{\frac{1}{2}}sign\left(e_{1}\right)\\ {\dot{e}}_{2}=\chi -\xi_{2}sign\left(e_{1}\right) \end{array} \end{array}$$

Through defining $\delta_{i}={\left[{\delta_{i,1}}^{T},{\delta_{i,2}}^{T}\right]}^{T}$, $\delta_{i,1}=|e_{1}{|}^{\frac{1}{2}}sign\left(e_{1}\right)$, δ_*i*, 2_ = *e*_2_, it can be derived that

(21) $$\begin{array}{r} \{\begin{array}{c} {\dot{\delta }}_{i,1}=-\frac{\xi_{1}}{2}{\left|e_{1}\right|}^{-1}e_{1}+\frac{1}{2}{\left|e_{1}\right|}^{-\frac{1}{2}}e_{2}\\ {\dot{\delta }}_{i,2}=-\xi_{2}{\left|e_{1}\right|}^{-1}e_{1}+\chi \end{array} \end{array}$$

Then, we define a positive definite matrix $\eta_{1}=\frac{1}{2}\left[\begin{array}{cc} 4\xi_{2}+{\xi_{1}}^{2} & -\xi_{1}\\ -\xi_{1} & 2 \end{array}\right]$ and introduce the Lyapunov function as:

(22) $$\begin{array}{r} V_{i}={\delta_{i}}^{T}\eta_{1}\delta_{i} \end{array}$$

We introduce $\eta_{2}=\frac{\xi_{1}}{2}\left[\begin{array}{cc} 2\xi_{2}+{\xi_{1}}^{2}-\frac{2\nu \left(\xi_{1}+1\right)}{\xi_{1}} & -\xi_{1}\\ -\xi_{1} & 1-\frac{2\nu }{\xi_{1}} \end{array}\right]$ and take the time derivative of *V*_*i*_:

(23) $$\begin{array}{l} {\dot{V}}_{i}\leq -2\xi_{1}{\left|e_{1}\right|}^{-\frac{1}{2}}\left[\left({\xi_{1}}^{2}+2\xi_{2}\right){\delta_{i,1}}^{2}-2\xi_{1}\delta_{i,1}\delta_{i,2}+{\delta_{i,2}}^{2}\right]\\ +\;2\xi_{1}{\left|e_{1}\right|}^{-\frac{1}{2}}\left(2{\delta_{i,1}}^{2}+2{\xi_{1}}^{-1}{\delta_{i,1}}^{2}+2{\xi_{1}}^{-1}{\delta_{i,2}}^{2}\right)\nu \\ =-2\xi_{1}{\left|e_{1}\right|}^{-\frac{1}{2}}{\delta_{i}}^{T}\eta_{2}\delta_{i} \end{array}$$

It can be found that ${\dot{V}}_{i}$ is a negative definite in the case that η_2_ is a positive definite. We can then obtain

(24) $$\begin{array}{r} \{\begin{array}{c} \xi_{1}>2\nu \\ \xi_{2}>\frac{{\xi_{1}}^{2}}{\xi_{1}-2\nu }\nu +\frac{\xi_{1}+1}{\xi_{1}}\nu \end{array} \end{array}$$

Based on Lyapunov stability theory, we can obtain $|e_{1}{|}^{\frac{1}{2}}sign\left(e_{1}\right)\to 0$ and *e*_2_ → 0. In this case, the estimate errors *e*_1_, *e*_2_ will converge to zero.

### 3.4. UAV Controller Approach

**Figure 3:**
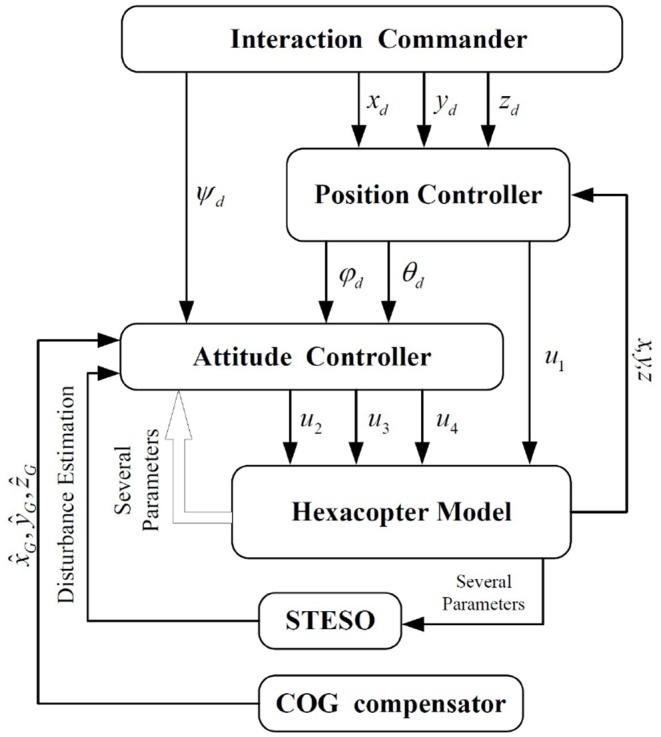
Control schematic of the overall hexacopter UAV system.

The hexacopter, whose rotational motion is coupled with translational motion, is difficult to control to perfection. In Figure 3, a control scheme is presented that improves the stability of the system. The control system is cascaded, being composed of two stages, namely the position controller and attitude controller. At the start of the control process, the desired positions (*x*_*d*_, *y*_*d*_, *z*_*d*_) will be sent to the position controller, which will then generate the desired attitudes (φ_*d*_, θ_*d*_) and transmit them to the attitude controller. The outputs of the attitude controller, which is responsible for guaranteeing that the attitudes track the desired orientations in a finite time, are the desired actuation forces generated by the hexacopter propellers. In addition, a COG compensator is incorporated to work out the real COG and transmit it to the whole control system.

In this section, a traditional PID controller is built for position control. It is only used to generate desired attitudes at translational directions, and the UAV will get to the desired position if the actual attitudes can track the desired orientations in a finite time.

#### 3.4.1. Attitude Control

In order to achieve high-precision attitude tracking in the presence of wind gusts and model uncertainties, some parameters should first be defined. ${\text{q}}_{\text{d}}={\left[q_{d0},{\text{q}}_{\text{d}\text{v}}\right]}^{T}$ and $\omega_{\text{d}}={\left[\omega_{dx},\omega_{dy},\omega_{dz}\right]}^{T}$ represent the attitude and desired angular velocities, respectively. We can then obtain the tracking error vector of the angular velocities $\omega_{\text{e}}={\left[\omega_{ex},\omega_{ey},\omega_{ez}\right]}^{T}$ as:

(25) $$\begin{array}{r} \omega_{\text{e}}=\omega -{\text{C}}_{\text{d}}^{\text{b}}\omega_{\text{d}} \end{array}$$

We take the time derivative of **ω_*e*_** and substitute Equations (5), (12), and (25) into ${\dot{\omega }}_{e}$:

(26) $${\dot{\omega }}_{\text{e}}=\text{S}(\omega_{\text{e}}){\text{C}}_{\text{d}}^{\text{b}}\omega_{\text{d}}-{\text{C}}_{\text{d}}^{\text{b}}{\dot{\omega }}_{\text{d}}-{\text{J}}^{-1}\text{S}(\omega )\text{J}\omega +{\text{J}}^{-1}\text{u}-\text{m}{\text{J}}^{-1}\text{c}+{\text{J}}^{-1}\text{d}$$

Also, the dynamics of the attitude tracking error can be obtained according to Equations (6), (7), and (25):

(27) $${\dot{\text{q}}}_{\text{e}}=\frac{1}{2}{\text{q}}_{\text{e}}⊗\left[\begin{array}{c} 0\\ \omega_{\text{e}} \end{array}\right]=\frac{1}{2}\left[\begin{array}{c} -\;{\text{q}}_{\text{e}\text{v}}^{\text{T}}\\ \text{S}({\text{q}}_{\text{e}\text{v}})+q_{e0}{\text{I}}_{3} \end{array}\right]\omega_{\text{e}}$$

The STESO-based backstepping controller for the attitude tracking controller is then developed with reference to Shi et al. (2018a). The backstepping is a very good fit for the cascaded structure of the UAV dynamics. To insure that the attitude tracking error ***q*_*e*_** converges to zero, we define the Lyapunov function as:

(28) $$\begin{array}{r} V_{A1}={q_{e\text{v}}}^{T}q_{e\text{v}}+{\left(1-q_{e0}\right)}^{2} \end{array}$$

We take time derivative of *V*_*A*1_:

(29) $${\dot{V}}_{A1}=2{\text{q}}_{\text{e}\text{v}}{\;}^{\text{T}}{\dot{q}}_{\text{e}\text{v}}-2(1-q_{e0}){\dot{q}}_{e0}={\text{q}}_{\text{e}\text{v}}{\;}^{\text{T}}\omega_{\text{e}}$$

By introducing a virtual control **ω_*ed*_** = −**M_1_*q*_*ev*_**, in which **M_1_** is the gain matrix of the controller, when the angular velocity tracking error **ω_*e*_** is equal to **ω_*ed*_**, we can obtain:

(30) $$\begin{array}{r} {\dot{V}}_{A1}=-{q_{e\text{v}}}^{T}{\text{M}}_{1}q_{e\text{v}}\leq 0 \end{array}$$

(31) $${\tilde{\omega }}_{\text{e}}=\omega_{\text{e}}+{\text{M}}_{1}{\text{q}}_{\text{e}\text{v}}$$

We then choose the Lyapunov function as:

(32) $$\begin{array}{r} V_{A2}=V_{A1}+\frac{1}{2}{{\tilde{\omega }}_{\text{e}}}^{T}J{\tilde{\omega }}_{\text{e}}+V_{x}+V_{y}+V_{z} \end{array}$$

We take the time derivative of *V*_*A*2_ according to Equations (25), (30), and (31):

(33) $$\begin{array}{l} {\dot{V}}_{A2}={\text{q}}_{\text{e}\text{v}}{\;}^{\text{T}}{\tilde{\omega }}_{\text{e}}-{\text{q}}_{\text{e}\text{v}}{\;}^{\text{T}}{\text{M}}_{1}{\text{q}}_{\text{e}\text{v}}+{\tilde{\omega }}_{\text{e}}^{\text{T}}(\text{J}{\dot{\omega }}_{\text{e}}+\text{J}{\text{M}}_{1}{\dot{\text{q}}}_{\text{e}\text{v}})+{\dot{V}}_{x}\\ +\;{\dot{V}}_{y}+{\dot{V}}_{z}=-{\text{q}}_{\text{e}\text{v}}{\;}^{\text{T}}{\text{M}}_{1}{\text{q}}_{\text{e}\text{v}}+{\tilde{\omega }}_{\text{e}}^{\text{T}}(\text{J}(\text{S}(\omega_{\text{e}}){\text{C}}_{\text{d}}^{\text{b}}\omega_{\text{d}}-{\text{C}}_{\text{d}}^{\text{b}}{\dot{\omega }}_{\text{d}})\\ -\text{S}(\omega )\text{J}\omega +\text{u}-m\text{c}+\text{d}+\text{J}{\text{M}}_{1}{\dot{q}}_{\text{e}\text{v}}+{\text{q}}_{\text{e}\text{v}})+{\dot{V}}_{x}+{\dot{V}}_{y}+{\dot{V}}_{z} \end{array}$$

By introducing the control input vector ***u***:

(34) $$\begin{array}{l} \text{u}=-\text{J}\left(\text{S}(\omega_{\text{e}}){\text{C}}_{\text{d}}^{\text{b}}\omega_{\text{d}}-{\text{C}}_{\text{d}}^{\text{b}}{\dot{\omega }}_{\text{d}}\right)+\text{S}(\omega )J\omega +m\text{c}-\text{J}{\text{M}}_{1}{\dot{q}}_{ev}\\ \;-{\text{q}}_{ev}-{\text{M}}_{2}{\tilde{\omega }}_{e}-\hat{\text{d}} \end{array}$$

${\dot{\text{V}}}_{A2}$ can be obtained by in substituting ***u***.

(35) $$\begin{array}{l} {\dot{V}}_{A2}=-{\text{q}}_{\text{e}\text{v}}{\;}^{\text{T}}{\text{M}}_{1}{\text{q}}_{\text{e}\text{v}}-{\tilde{\omega }}_{\text{e}}^{\text{T}}{\text{M}}_{2}\tilde{\omega }+{\tilde{\omega }}_{\text{e}}^{\text{T}}\tilde{\text{d}}+{\dot{V}}_{x}+{\dot{V}}_{y}+{\dot{V}}_{z}\\ \leq -\lambda_{\min}({\text{M}}_{1}){\left\|{\text{q}}_{\text{e}\text{v}}\right\|}^{2}-\lambda_{\min}({\text{M}}_{2}){\left\|{\tilde{\omega }}_{\text{e}}\right\|}^{2}+\left\|{\tilde{\omega }}_{\text{e}}\right\|\left\|\tilde{\text{d}}\right\|\\ -\lambda_{\min}(\eta_{1})({\left\|\delta_{\text{x}}\right\|}^{2}+{\left\|\delta_{\text{y}}\right\|}^{2}+{\left\|\delta_{\text{z}}\right\|}^{2})\\ \leq -\lambda_{\min}({\text{M}}_{1}){\left\|{\text{q}}_{\text{e}\text{v}}\right\|}^{2}-\lambda_{\min}({\text{M}}_{2}){\left\|{\tilde{\omega }}_{\text{e}}\right\|}^{2}+\left\|{\tilde{\omega }}_{\text{e}}\right\|\left\|\delta \right\|\\ -\lambda_{\min}(\eta_{1})({\left\|\delta_{\text{x}}\right\|}^{2}+{\left\|\delta_{\text{y}}\right\|}^{2}+{\left\|\delta_{\text{z}}\right\|}^{2})\\ \leq -\lambda_{\min}({\text{M}}_{1}){\left\|{\text{q}}_{\text{e}\text{v}}\right\|}^{2}-(\lambda_{\min}({\text{M}}_{2})-\frac{1}{2}){\left\|{\tilde{\omega }}_{\text{e}}\right\|}^{2}\\ -(\lambda_{\min}(\eta_{1})-\frac{1}{2}){\left\|\delta \right\|}^{2} \end{array}$$

where $\left\|\tilde{\text{d}}\right\|=\left\|\delta_{2}\right\|\leq \left\|\delta \right\|$, $||{\tilde{\omega }}_{e}||||\delta ||\leq \frac{1}{2}\left(||{\tilde{\omega }}_{e}|{|}^{2}+||\delta |{|}^{2}\right)$, ${\left\|\delta \right\|}^{2}={\left\|\delta_{\text{x}}\right\|}^{2}+{\left\|\delta_{\text{y}}\right\|}^{2}+{\left\|\delta_{\text{z}}\right\|}^{2}$, and λ_min_(***M***) denotes the minimal eigenvalue of ***M***. Thus, ${\dot{V}}_{A2}\leq 0$ whenever $\lambda_{\min}(\eta_{1}),\lambda_{\min}({\text{M}}_{2})\geq \frac{1}{2}$. In that case, it can be concluded that the attitude error ***q*_*e*_**, angular velocity tracking error **ω_*e*_**, and estimation errors **δ_*x*_**,**δ_*y*_**,**δ_*z*_** would be uniformly ultimately bounded and exponentially converge to zero.

#### 3.4.2. COG Compensation System

As shown in Figure 1, positional variety in the COG of the vehicle will occur when the UAV conducts tasks that involve the motion of the robotic arm. The dynamic model of the whole system will then be changed during the flight referring to Equation (10). Additionally, the stability of the UAV will be impacted. To overcome this problem, a COG compensation system, which will not be shown here due to the limitations of article length but is detailed in our previous work Jiao et al. (2018), can be implemented. However, the real COG cannot be calculated accurately through this system due to several measuring errors. It will also play a part in the model uncertainties included by the lumped disturbance, which will be estimated by the STESO.

### 3.5. Interaction Between UAV and Human

#### 3.5.1. Interaction Regulation From Human to UAV

**Figure 4:**
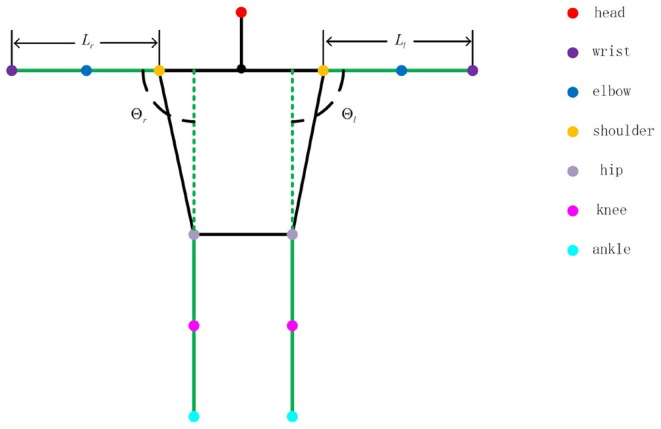
Prototype of human joint.

An interaction regulation scheme from human to UAV is developed in this section using the human pose. According to the given interaction regulation, the UAV can be attracted by a distant human by their holding a constant pose, which should last more than 5 s, to initiate the interaction. After the interaction initialization is completed, an LED will begin flashing as feedback to the human. Moreover, both target flight direction and distance commands can be communicated to the UAV in a very simple and direct way through human pose changes. As depicted in Figure 4, some given straight lines compose a nonobjective human, in which the colorized points, representing the joints of the human body, can be detected and signed by the pose estimation system mentioned in section 3.1. The whole interaction regulation scheme is based on the coefficients (*L*_*l*_,*L*_*r*_,Θ_*l*_ and Θ_*r*_) given in Figure 4. The meanings of different combinations of coefficient values are listed in Table 1.

**Table 1:** Meanings of different coefficient combinations.

**Combination**	**Meaning**
80 < Θ_*l*_ < 100 and 80 < Θ_*r*_ < 100	Interaction initiation
100 < Θ_*l*_ < 150 and *L*_*r*_ < *L*_*l*_	Flight direction command 1: right front with respect to the human
30 < Θ_*l*_ < 80 and *L*_*r*_ < *L*_*l*_	Flight direction command 2: right rear with respect to the human
100 < Θ_*r*_ < 150 and *L*_*l*_ < *L*_*r*_	Flight direction command 3: left front with respect to the human
30 < Θ_*r*_ < 80 and *L*_*l*_ < *L*_*r*_	Flight direction command 4: left rear with respect to the human
100 < Θ_*l*_ < 180 and 100 < Θ_*r*_ < 180	Flight distance command: based on positions of two wrist joints
0 < Θ_*l*_ < 30 and 0 < Θ_*r*_ < 30	End flag

**Table:** **Algorithm 1**. The Whole Interaction Procedure.

1: Execute human pose estimation system initially and record video from camera;
2: **if** Detect interaction initiation action for more than 5 s **then**
3: Measure depth information of the detected human and approach him immediately;
4: Continue to execute human pose estimation system;
5: **if** interaction initiation **then**
6: **if** Detect flight direction command **then**
7: Record target flight direction;
8: **if** Detect flight distance command **then**
9: Record target flight distance;
10: **if** End flag **then**
11: Execute flight command from human and return to the first line in the procedure;
12: **end if**
13: **end if**
14: **end if**
15: **end if**
16: **else**
17: Continue execute human pose estimation system and record the video from camera;
18: **end if**

**Figure 5:**
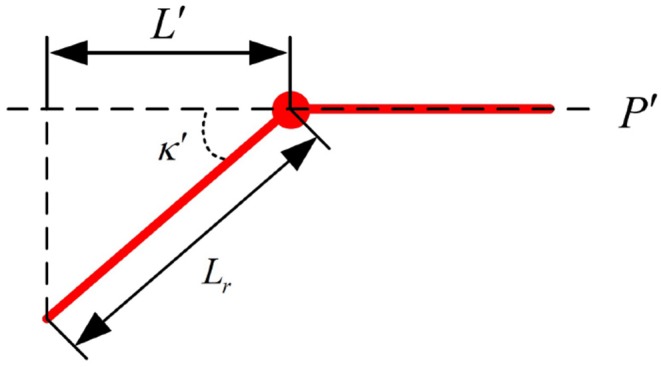
Aerial view of nonobjective human.

Specifically, the human who is executing search tasks in the field can attract the UAV for search assistance by conducting the interaction initiation action, keeping parallel to the UAV camera, for more than 5 s until the UAV responds by flashing its LED. Moreover, to control the UAV more easily and intuitively, the target command flight direction is just parallel to the human arm, and the target command flight distance is based on the distance between the two wrist joints. As shown in Figure 5, the particular flight direction and distance command methods are given, and a nonobjective aerial view of the human, which represents flight direction command 1 mentioned in Table 1, is provided. The two red straight lines are human arms. We can easily obtain the target flight direction with respect to plane *P*′, which is parallel to the UAV camera plane:

(36) $$\begin{array}{r} \kappa^{\prime }=\arccos\frac{L^{\prime }}{L_{r}} \end{array}$$

The case with other flight direction commands is similar to that mentioned above. Additionally, the target flight distance command transmitted to the UAV is proportional to the distance between two detected wrist joints. The constant length of a fully stretched human arm, *L*_*l*_ in the picture, represents the unit used as a reference for the distance command. The unit depends on the character of the performed task and would be defined in advance. Moreover, the interaction procedure in the automated exploration task is given in Algorithm 1.

#### 3.5.2. Communication From UAV to Human

As shown in Figure 8, a vertical column of RGB LEDs, which are used to communicate the state and intents of the UAV to the user as feedback, are fixed on the left undercarriage of the hexacopter. It is controlled by a combination of a pixhawk and an STM32-based board with three colors (red, blue, and green). By changing the color and flicker frequency of the RGB LEDs through the communication regulation formulated in advance, the state and intents of the UAV can be transmitted to the user.

## 4. Simulation Results and Discussion

To demonstrate the validity and performance of the proposed STESO and corresponding control scheme, several simulations of attitude tracking under external disturbance torque will be conducted using a MATLAB/SIMULINK program with a fixed-sampling time of 1 ms in this section. As a contrast, a traditional second-order ESO is built combined with the proposed attitude controller in the same simulation progress. In addition, we assume that the three-axis components of the external disturbance torques exerted on the UAV are the same and that one of them can be described as:

(37) $$\begin{array}{l} d=0.9\sin\left(2.5\pi t-1\right)+1.2\sin\left(2\pi t+2\right)+1.95\sin\left(0.3\pi t\right)\\ \;+0.45\left(0.2\pi t+6\right)+0.15\sin\left(0.1\pi \right)\\ \;+0.75\sin\left(0.05\pi -3.5\right)+1.05\sin\left(\pi t-0.9\right)\\ \;+1.5\sin\left(0.01\pi +1\right)-1.185 \end{array}$$

Moreover, the dynamic model built in section 2.3 is taken as the basis of the simulation of the proposed observer and controllers. The simulation parameters, which are verified to be very close to the reality of the single multi-copter and are listed in Table 2, are generated by the online toolbox of Quan (2018). Although this is a list of coefficients for a single multi-copter without a robotic arm, it is also useful in our simulation system, as the rest of the model uncertainty can also be included in the lumped disturbance and estimated by the proposed observer. Additionally, we choose the BSC gains as ***M*_1_** = 10***I*_3_**, ***M*_2_** = 3***I*_3_**, the STESO gains as ξ_1, *i*_ = 28, ξ_2, *i*_ = 58, and the traditional second-order ESO gains as ***L*** = [35 380 600]^*T*^.

**Table 2:** Coefficients in the simulation system.

***Coefficients***	***Particulars***	***Value***
*m*	Mass of the whole UAV system	10.5 kg
*J*_*x*_	Roll inertia	4.557 × 10^−1^*kg*·*m*^2^
*J*_*y*_	Pitch inertia	4.557 × 10^−1^*kg*·*m*^2^
*J*_*z*_	Yaw inertia	7.724 × 10^−1^*kg*·*m*^2^
*l*	Motor moment arm	0.5 m

**Figure 6:**
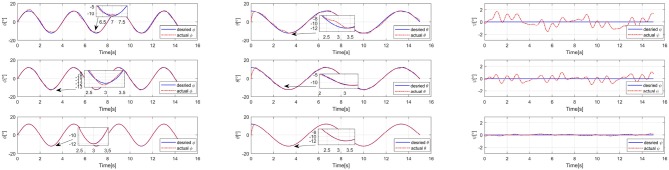
Simulation results of roll, pitch, and yaw angle-tracking with unknown external torque disturbance. Without observer (Up), traditional ESO (Middle), and STESO (Down).

It can be seen from Figure 3 that an effective attitude controller is the foundation of UAV motion and needs to work well during a UAV exploration task with unknown external disturbances. As shown in Figure 6, several attitude tracking simulations have been conducted based on the proposed back-stepping controller with the STESO and traditional ESO. The desired attitude references are given as:

(38) $$\begin{array}{r} \Theta_{d}={\left[12\text{ sin}(0.5\pi t)\;12\text{ cos}(0.3\pi t)\;0\right]}^{T}\text{ deg} \end{array}$$

**Figure 7:**
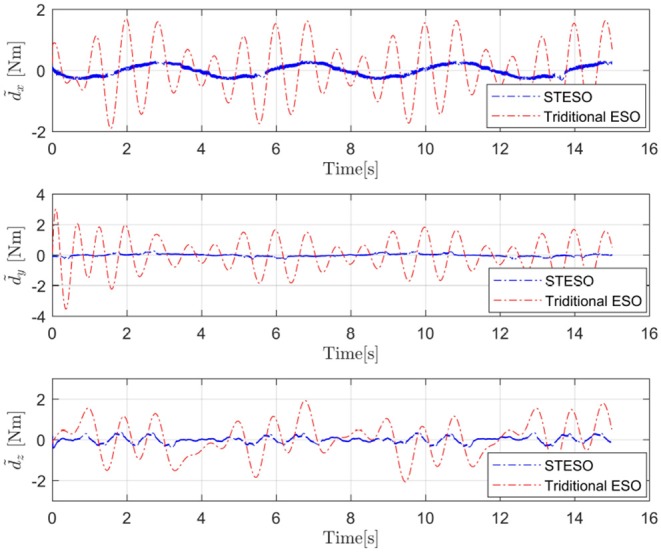
Disturbances estimate errors (${\tilde{d}}_{x},{\tilde{d}}_{y},{\tilde{d}}_{z}$) of the STESO and traditional ESO.

From these figures, we can easily determine that the tracking errors are largest in all of the channels (roll, pitch, and yaw) without any observers. The tracking trajectories are influenced seriously. Moreover, we also find that it is obviously improved with observers to estimate and then attenuate the disturbance directly, even though the disturbance is not estimated completely. Compared to being equipped with the traditional ESO, the tracking error is also further reduced by using the STESO. Further, the disturbance estimate errors of the STESO and traditional ESO in all channels are shown in Figure 7, showing that the STESO could make a better estimation than the traditional ESO. Thus the UAV can attain better attitude tracking performance under the control of the proposed controller with an STESO.

## 5. Experimental Results and Discussion

This section details several experiments, including hovering with wind gusts and a synthetic interaction experiment between humans and a UAV, that were conducted in a playground to validate all the above-mentioned theories.

### 5.1. Hardware Platform

**Figure 8:**
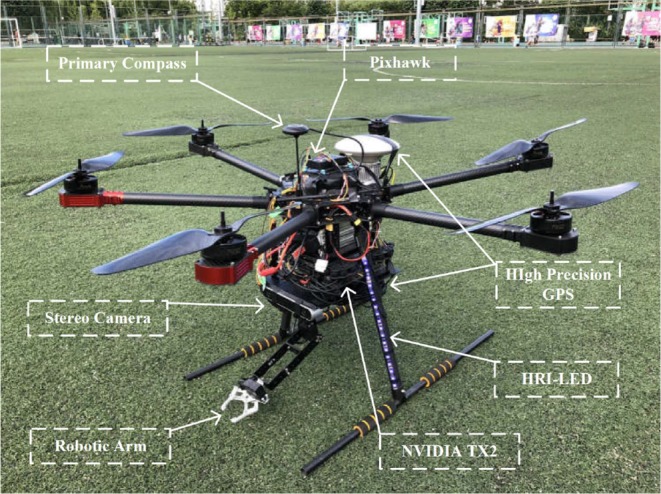
Prototype of the proposed interactive UAV.

Figure 8 shows the UAV platform suitable for our interaction system that was constructed. It is a hexacopter with a 143-cm tip-to-tip wingspan, six 17-inch propellers, a height of 58 cm, and a total mass of 10.5 kg including the robotic arm, which is fixed under the vehicle. Each rotor offers lift force of up to 4.0 kg, which is enough for the whole system. In addition, Open-source PIXHAWK hardware (Meier, 2012), which includes an STM32 processor and two sets of IMU sensors, is fastened to the top of the UAV and is used for sensor data integration, attitude computation, mode switching, state assistant feedback, controller and STESO operation, emergency security protection, etc. Moreover, an NVIDIA TX2 equipped with six CPU cores and 256 CUDA cores is utilized in the interaction system in which the human pose estimation and depth computation tasks are loaded. A binocular stereo camera, which offers 720P video transmission of up to 60 fps, is placed at the front of the vehicle to obtain the three-dimensional position of the target with respect to the UAV. Additionally, to ensure the safety of the experimental partner during close-range interaction, a high-precision GPS is utilized to supply accurate information on the absolute and relative position of the vehicle, which can also enable stable UAV hovering. Moreover, the vehicle uses HRI-LEDs to communicate its state and intents to the user, and a compass is placed at the highest point of the UAV to prevent electromagnetic interference.

### 5.2. Hovering With Wind Gusts

**Figure 9:**
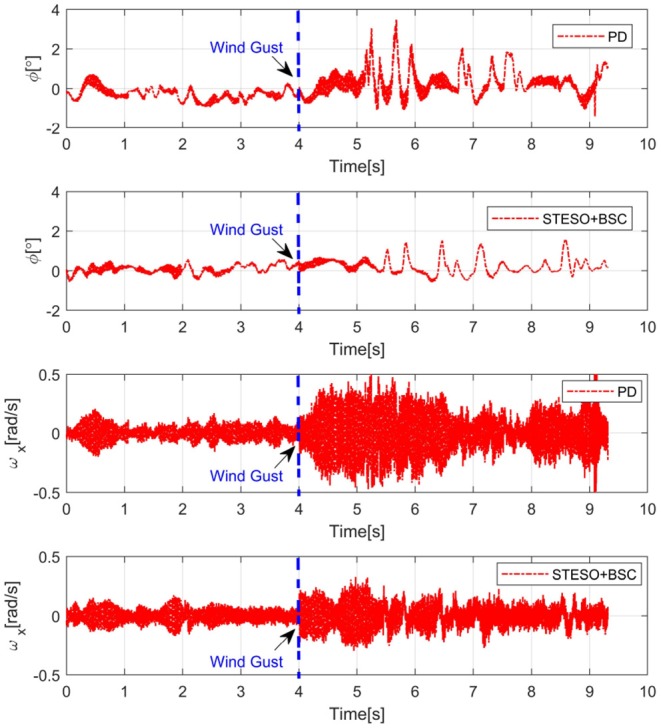
Experimental curves of attitude angle φ and angular rate ω_*x*_ during hovering with wind gusts.

To demonstrate the performance of the developed method for a UAV subject to lumped disturbances including wind gusts and model uncertainties, a hovering experiment was conducted with wind disturbance generated by several electrical fans. We set the BSC gains at ***M*_1_** = *diag*(10, 10, 4) and ***M*_2_** = *diag*(0.2, 0.2, 0.28) and the STESO gains at ξ_1, *i*_ = 1.2, ξ_2, *i*_ = 0.3. The results for attitude angle φ and angular velocity ω_*x*_ under STESO-BSC and a traditional PD controller can be found in Figure 9. It can be observed that the chattering is markedly reduced under STESO-BSC compared to PD. In particular, the peak values of the attitude φ under PD and STESO-BSC are no more than 4° and 2°, respectively. Additionally, under control of PD, the UAV has more drastic chattering with angular velocity varying more quickly.

### 5.3. Human-UAV Interaction

**Figure 10:**
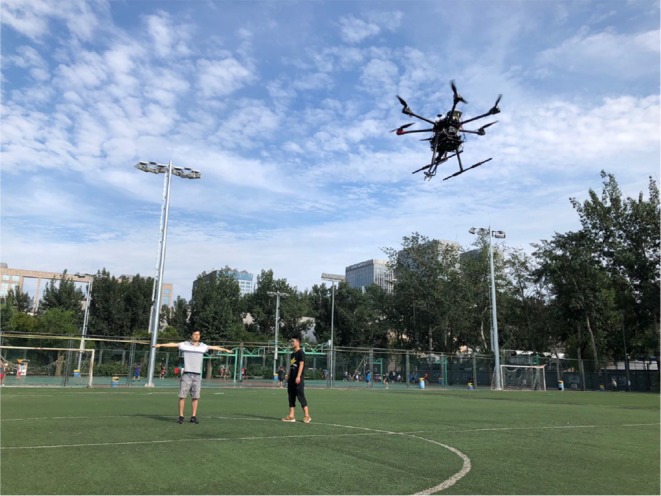
Interaction between human and UAV. Written informed consent for publication was obtained from the individuals in this image.

As shown in Figure 10, in this section, an automated exploration task with the proposed interaction system was conducted in a playground. It includes interaction initiation and particular commands, which contain UAV flight target direction and distance, communicated from human to UAV.

#### 5.3.1. Interaction Initiation

**Figure 11:**
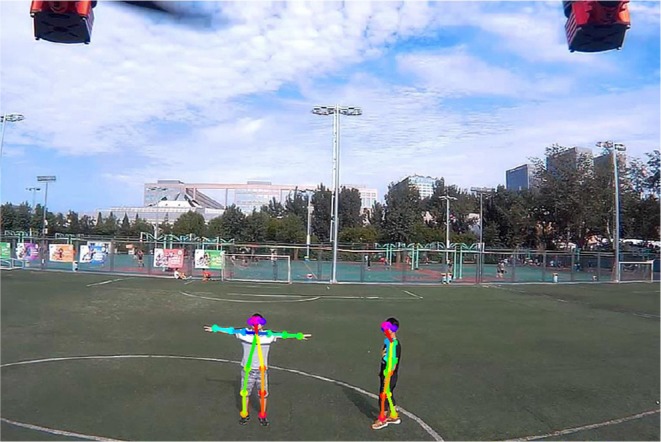
Interaction initiation. Written informed consent for publication was obtained from the individuals in this image.

As shown in Figure 11, the interaction initiation action of a human was detected by the UAV at a distance in the playground. Meanwhile, another person who walked around served as a disturbance term during the whole interaction initiation process. It had been verified that the UAV can discriminate these poses from human walking and other human motions. All the key joints of the human were obtained from the human pose estimation system. The average inference time per image is about 0.167 s. After the interaction initiation process was finished, the UAV approached human, while flashing its light as feedback, for further command information.

#### 5.3.2. Automated Exploration Task

**Figure 12:**
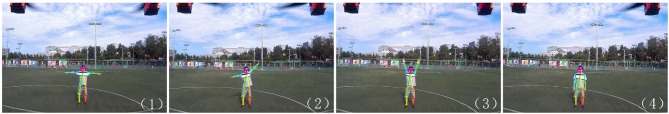
Target direction and distance communication to the UAV. (1) Beginning. (2) Target direction command. (3) Target distance command. (4) End. Written informed consent for publication was obtained from the individual in this image.

**Figure 13:**
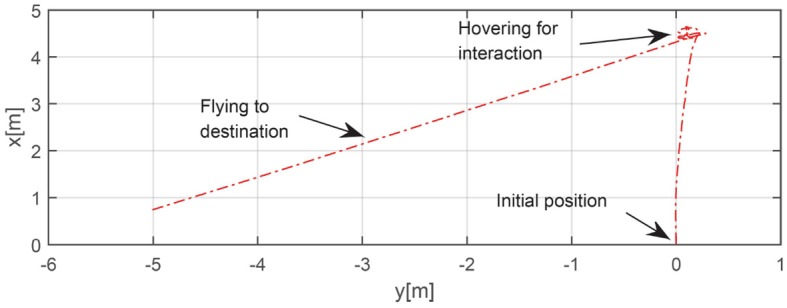
Trajectory in automated exploration task.

After the interaction initiation process was completed, the human received the UAV's feedback information and was ready to give the next command to the UAV. The specific steps of close interaction can be seen in Figure 12, in which parts (2) and (3) represent direction and distance commands, respectively, as outlined in section 3.5.1. According to the positions of the human joints and Equation (36), the target direction with respect to the camera plane was determined to be 36.5°. Meanwhile, using the reference unit for the distance command set in this experiment, 10 m, the final target distance was determined to be 17.4 m. The flight trajectory was recorded and is shown in Figure 13. The actual direction angle and distance are 35.4° and 16.9 m, respectively, the errors of which are small enough for field exploration. However, owing to the limitations of figure space, the trajectory to the destination is shown only partially. We could conclude from the experiment that the proposed interaction system is qualified to complete the field exploration task. However, through the whole experiment, we also found that the process of the interaction between UAV and human was not quick enough. As the frame-rate of pose estimation is still limited in spite of its improvement through our work, the human has to wait for a while for the response from the UAV at every step of the interaction, which will influence the interactive efficiency and experience. More attention should thus be paid to developing this state-of-art interaction technique in the future.

## 6. Conclusion

In this study, an intuitive end-to-end human-UAV interaction system, in which a UAV can be controlled to fly to a corresponding direction and distance by human poses, was built to assist in field exploration. Moreover, a real time multi-human pose estimation system, which performs with low latency while maintaining competitive performance, was built with which a human can communicate with the UAV under a proposed interaction regulation scheme. By introducing the super-twisting algorithm, an STESO was constructed and applied to the UAV attitude control system to estimate and attenuate complex disturbances, such as wind gusts, model uncertainties, etc. Based on the STESO, a back-stepping attitude controller was built that was proved through several simulations and experiments to have a better performance than a back-stepping controller with a traditional ESO. Finally, an integrated human-UAV interaction experiment was conducted in which the effectiveness of the whole system and its individual components were demonstrated.

## Data Availability Statement

The datasets generated for this study are available on request to the corresponding author.

## Ethics Statement

All procedures performed in studies involving human participants were in accordance with the ethical standards of the institutional and/or national research committee and with the 1964 Helsinki Declaration and its later amendments or comparable ethical standards. Informed consent was obtained from all individual participants involved in the study.

## Author Contributions

RJ conceived and designed the STESO and corresponding back-stepping attitude control algorithm. RJ, ZW, and RC built the human pose estimation system. YR constructed the depth estimation system. MD and WC assisted in the manuscript writing.

### Conflict of Interest

The authors declare that the research was conducted in the absence of any commercial or financial relationships that could be construed as a potential conflict of interest.
